# The Experiences of People Living With Chronic Pain During a Pandemic: “Crumbling Dreams With Uncertain Futures”

**DOI:** 10.1177/10497323211014858

**Published:** 2021-06-17

**Authors:** Kristina Amja, Marie Vigouroux, M. Gabrielle Pagé, Richard B. Hovey

**Affiliations:** 1 McGill University, Montreal, Quebec, Canada; 2 Montreal Children’s Hospital, Montreal, Quebec, Canada; 3 Centre de Recherche du CHUM, Montreal, Quebec, Canada; 4 Université de Montréal, Montreal, Quebec, Canada

**Keywords:** chronic, pain, qualitative, hermeneutics, quality of life Quebec, Canada

## Abstract

People living with chronic pain experience multiple challenges in their daily activities. Chronic pain is complex and often provokes life circumstances that create increased social isolation. Living with chronic pain during the pandemic may add additional layers of complexity to their daily lives. The researchers endeavored to explore the experiences of people living with chronic pain during the COVID-19 pandemic. Researchers conducted semi-structured, open-ended interviews about how the pandemic influenced participants’ lives. The interviews were recorded and analyzed using an applied philosophical hermeneutics approach. The findings were feeling socially isolated, losing their sense of livinghood, and experiencing augmented stress levels which, in most cases, aggravated their chronic pain. In addition to gaining an in-depth understanding of the needs of people living with chronic pain, these findings may guide policy decisions with the intention of improving health care access and the overall experiences of people living with chronic conditions during a pandemic.

## Introduction

Living with chronic pain is a multifaceted, complex, and often solitary experience (Furnes et al., 2015). One in four Canadians suffers from chronic pain (Shupler et al., 2019). Chronic pain is described as pain that lasts more than 3 to 6 months; it can arise at any age and may be detrimental in all aspects of one’s life (Merskey et al., 1994). People suffering from chronic pain are often not only limited in their capacity to participate in their day-to-day activities but also report effects on their relationships and suffering from social isolation (Furnes et al., 2015; Hovey et al., 2020).

The unique circumstances in which we currently live, such as the COVID-19 pandemic, potentially add a whole new layer of challenges for people living with chronic pain. Karos et al. (2020) explore the negative impacts of social changes prompted by the COVID-19 crisis especially for people living with chronic pain, such as threatening an individuals’ fundamental social needs for autonomy, belonging, and justice (Karos et al., 2020). Given this information, health care professionals are now more than ever encountering persons and their families experiencing this multidimensional challenge. Philip Strong, a known sociologist who studied pandemics, explains that a major outbreak of unknown can be accompanied by plagues of fear, panic, suspicion, and stigma; and pandemics expose the potential fragility of human social structure (Strong, 1990).

The intention of this study is to learn from our research participants about their experience of living with chronic pain during the COVID-19 pandemic. The preponderance of research to date has been primarily quantitative in nature or focused on health care professionals. Thus far, little attention has been given to the experiences of persons suffering from chronic pain during a life-altering pandemic. Several studies, such as Holmes et al., urge researchers to address the lived experience of people living through the COVID-19 pandemic (Holmes et al., 2020). The intent of our research project is to utilize philosophic hermeneutics in an exploration of the experience of living with chronic pain during a pandemic, rather than one focused on the biomedical aspects of explaining chronic pain (Hovey et al., 2018). Although we are aware that these findings may apply to other populations, the experiences described in this article were collected exclusively from people living with chronic pain. The extent of chronic pain was not the concern of this research, but rather we focused on the meaning of the lived experience of pain while managing it during the COVID-19 pandemic.

## COVID-19 in Quebec

All the participants in this study are part of the Quebec Low Back Pain Consortium, an organization that conducts research on local populations with low-back pain. As such, all the participants in our study were Quebec residents (Pagé et al., 2020). This fact matters because Quebec had a particularly severe “first wave” of COVID-19, starting in March 2020. As of October 7, 2020, 5,907 people had died from COVID-19 in the province, compared with 9,541 in all of Canada. Quebec’s population accounts for 22.56% of the country, and yet represented 61.91% of its total COVID-19-related deaths (Gouvernement du Québec, 2020; Government of Canada, 2020; Statistics Canada, 2020). On March 12, 2020, the Quebec provincial government closed all schools, physical activity facilities, and non-essential businesses for 1 day. This closure was then prolonged for 2 weeks, and finally until further notice, thereby leaving the population in a limbo of uncertainty. The Premier held a daily press conference with the provincial Public Health Director and the Minister of Health. In addition, the Canadian army was called in to aid health care workers in the government-run residential and long-term care centers. This contextualization is necessary to understand the situation in which the Quebec population found itself during the “first wave” of the pandemic at which time we collected our data. During this “first wave” in Quebec, the health care resources were still not adapted to the reality of the pandemic; for example, telehealth was not readily available.

This qualitative research project focuses on the experience of persons living with chronic pain during the COVID-19 pandemic using philosophical hermeneutics. We were curious about the effects of this complex experience on people’s day-to-day lives while living with chronic pain under the influence of COVID-19.

## Research Methodology and Design

### Participants

The study was approved through the McGill University Research Ethics Board (IRB A06-B31-13A). Inclusion criteria required the participants to (a) be part of the Quebec Low Back Pain Consortium (QLBPC) cohort study; (b) have completed the COVID-19 survey sub-project from the QLBPC cohort study and agreed to be contacted for a follow-up interview; (c) have lived with chronic pain for at least 1 year, and (d) speak French or English. The QLBPC is an initiative of the Quebec Pain Research Network, which aims to further chronic pain research in the province of Quebec (Quebec Pain Research Network, 2021a). The QLBPC maintains a database of people self-reporting lumbar back pain and sends invitations to participate in research studies to those people in the database who have consented to be contacted (Quebec Pain Research Network, 2021b). In the spring of 2020, the QLBPC initiated a survey on the effects of COVID-19 on people living with lumbar pain, from which emerged the need for a qualitative inquiry. This article only outlines the details of the qualitative inquiry.

**Figure 1.**
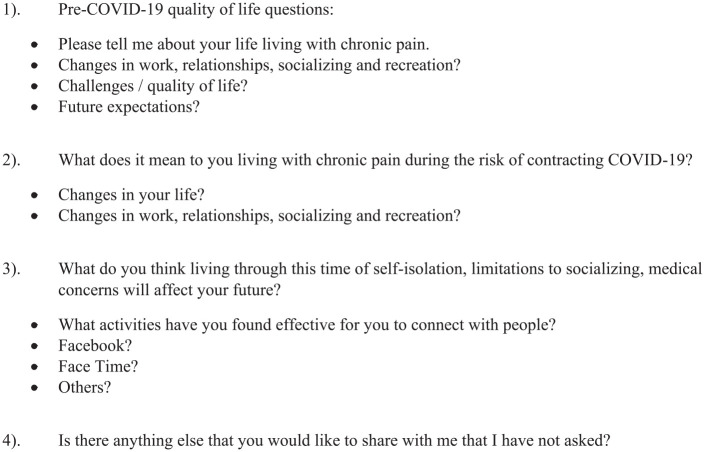
Open-ended semi-structured interview guide.

Participants were recruited through email and phone, using the QLBPC’s database to identify the people who had already participated in the COVID-19 survey and agreed to be contacted for a follow-up interview. Twenty-two participants (10 men and 12 women) were individually interviewed using open-ended questions in a semi-structured interview guide (Figure 1). All participants were between their mid-twenties and late sixties in age and had lived with chronic low-back pain for at least 1 year (Table 1). Interviews lasted 37.27 minutes on average, with the shortest one being 18 minutes and the longest one, 63 minutes.

**Table 1.** Demographic Information.

	Overall
Participants’ characteristics	(*n* = 22)
Age	
*M* (*SD*)	49.3 (11.1)
Median [Min, Max]	49.0 [26.0, 67.0]
Sex	
Female	12 (54.5%)
Male	10 (45.5%)
Pain duration	
>5 years	16 (72.7%)
1–5 years	6 (27.3%)

### Methodology/Approach

The interviews were audio-recorded, transcribed into text, and analyzed interpretively according to philosophical hermeneutic tradition as outlined by Moules (2015). The participants’ transcripts were anonymized to ensure confidentiality. The interviews conducted in French were translated to English by the authors. Emerging interpretations were reinforced with relevant chronic pain literature and the philosophical principles of hermeneutical research. We endeavored to gain an in-depth understanding of the experience of living with chronic pain during a pandemic. Specifically, this study was guided by Hans-Georg Gadamer’s tenets of philosophical hermeneutics:

Gadamer’s dialogical philosophical hermeneutics, enlists the view that the purpose of philosophical hermeneutics is to unite the consciousness of one subject with that of the others. This is referred to as appropriation in which the researcher is working toward understanding the experience of the individual within the context of a community of experiences. (Kearney, 2003)

The application of a hermeneutic approach was supported following the methodological procedure guided by Moules (2015). Unlike quantitative and some qualitative approaches, applied philosophical hermeneutics do not rest upon strict methodology to yield findings (Moules, 2015). Instead, hermeneutics require “conversations through in-depth, rigorous, reflexive, and communal attention to the data” with which the team of researchers engages within the hermeneutical approach (Moules, 2015). The culmination of this intensive interpretive work was intended to extend, disrupt, and transform our pre-understandings of the topic (Kearney, 2003). The rigorous process of going through this narrative is based on the understanding of the philosophy of Gadamerian hermeneutics and is associated with the researchers’ experience in reflexivity. Given that reflexivity requires an awareness of the researcher’s contribution, pre-understandings and experiences contribute to the construction of meanings throughout the research process.

### Applied Philosophical Hermeneutics

Gadamer, a philosophical hermeneutic scholar, referred to hermeneutics as the “art of understanding” (Gadamer, 1996). Understanding is a transformative and reflective practice, which may incite new ideas and perspectives within our comprehension of a topic (Davey, 2006; Grondin, 2002). A collective understanding results from each voice within a topic, including the researchers’, leading to historical situatedness that is contextually and ontologically driven. Philosophical hermeneutics is concerned with how these experiences play out and manifest in our research participants’ lives. In other words, how the participants navigate their new life circumstances (Davey, 2006).

The process from a transcribed interview to an interpretation of value and resonance that can extend or overturn understanding of a topic is challenging to articulate (Hovey, 2006). The metaphor of the fusion of horizons can be of assistance in looking between the whole and the parts. In Gadamerian hermeneutics, a fusion of horizons refers to a communicative process that attempts to make meaning of our world and other people’s perspectives about a topic. The communicative process informs our point of view, perspective, or worldview. Our individual perspective of the world evolves throughout our lifetime, during enculturation as people, history, and education with many other factors that have contributed to our personhood. The person who is trying to authentically understand another’s perspective is open to a fusion of diverse experiences, worldviews, and understandings to create a newly expanded horizon (Hovey et al., 2017). It invites a consideration of the particulars of a topic in the context of the familiar, while also attending to the familiar in the context of the particulars (Gadamer, 1989; Grondin, 2002; Moules, 2002). As hermeneutic researchers, we engage the lived experience of living with chronic pain during a pandemic and explore these experiences through a philosophical hermeneutical approach (Gadamer, 1989).

In this study, researchers individually read the interview transcripts and identified the most salient findings. After group discussion, certain findings were then further developed, through re-reading of the transcripts, to identify other data that expanded the topic. This movement in and out of the data allowed for consideration of findings that might not have been initially visible, bringing them to the larger context of shared experiences (Moules, 2015). Hermeneutic research is at its core about discussion, reflection, and interpretation; therefore, the authors met weekly to discuss and confront their self-awareness and biases pre-, during, and post-research to ensure that reflexivity was addressed as an ongoing process.

## Findings

### Social Isolation Versus Social Connectedness

A definition of social isolation is provided below to help inform the readers:

Isolation is a form of loss. What is lost in it is nearness to others. In the experience of isolation, there seems to be suffering along with solitude. [. . .] Solitude is not always suffering. (Gadamer, 1998)

Whereas a definition for social connectedness is the following:

[. . .] Social connectedness is defined as the sense of belonging and subjective psychological bond that people feel in relation to individuals and groups of others. (Haslam et al., 2015)

### Social Isolation

Isolation means something other than solitude (Gadamer, 1998). With solitude, one has a choice. For example, going for a long walk to get away from everyone to clear one’s head, with the possibility to return to meet up with people later. On the contrary, isolation from chronic pain is lived with a certain degree of suffering: one is isolated from people and does not have people to connect with and return to. Consequently, as explained by Hovey et al., when everything in one’s life changes due to chronic pain, when nothing is as it used to be, one can feel exhausted, shattered, isolated, and does not know what to do anymore as their social network diminishes; one hides away from the world. Isolation creates an additional layer of pain leading to depression, despondency, feelings of loss, purpose, and personal value (Hovey et al., 2020).

One of the most salient findings that emerged from this study was a change in the familiar patterns of social connectedness of the participants. Social distancing measures put into place to slow the spread of COVID-19 in Quebec appears to have accentuated the isolation of people living with chronic pain. One living with chronic pain may already experience a deficit in social interaction and this becomes even more profound during a pandemic where there is forced isolation on an existing layer of isolation (Hovey et al., 2020).

One participant shared the following with us during their interview: “In terms of socializing with family and friends . . . I haven’t seen anyone in two months.” This participant, continuing to work as an Emergency Medical Technician during the confinement period, was adamant about avoiding contact with friends and family to reduce their risk of contracting the virus from him. He felt a great responsibility to his friends and family knowing that he was still in contact with strangers who might be affected by COVID-19 due to the nature of his work.

Another participant spoke about alternatives to in-person socialization:

[Socialization] pretty much disappeared. I took a walk in the park with a friend two meters apart about once a week, to keep in touch. Otherwise, all social interactions happened over phone, videoconference, WhatsApp, Skype, and the like. [Pause] It’s a lot more impersonal.

Through this quote, this participant expressed the limitations of virtual socialization. She felt that, while social media is a way to foster socialization in the current circumstances, it paled in comparison to meeting people in person and spending time with them in the same space. Being adaptable seems to be key to successfully enduring pain and pandemic, from person-to-person to screen-to-screen.

A third participant spoke of the anxiety to socialize due to their current medical conditions: “Obviously there’s extra stress in terms of interactions since I know very well that if I get COVID, I’ll be on the right-hand list.” The participant was referring to the list of people who had died because of contracting the virus, bluntly stated that socializing during a pandemic is a matter of life or death. This was certainly a sentiment shared by many other participants, to varying degrees. The added distress created by knowing that contracting COVID-19 could lead directly to one’s death can darken an already challenging lived experience of pain.

A fourth participant, interviewed once the regulations surrounding the lockdown had begun to loosen, expressed that even in-person meetings have changed: “My spouse’s brother came over and my daughter got close. I told her ‘you have to stay 2 meters away from your uncle.’ I took out the tape to show her. I didn’t think it was that far.” Socializing during a pandemic is complicated and not as enjoyable as before. Fear may have taken a toll on people’s mental health. Although fear and anxiety may be experienced in many aspects of being-in-the-world, living with a diagnosis of a serious illness forces the person into becoming patient and negotiating the significance of a heightened awareness of their life, health, and future.

### Social Connectedness

On the contrary, some participants expressed that the new ways of socializing that emerged during the confinement period allowed them to feel as connected to their loved ones as before. One participant explained,

Obviously, socializing was limited but I made sure to feel like I was surrounded. We’d have Zoom calls with friends all the time. We’d go outside at a distance in the park. I can feel things have changed but not that much.

Interestingly, this participant describes a similar experience to those described above, but her outlook is noticeably different. This difference in perception may be related to the socializing experiences and habits of each participant prior to confinement. It appears that having a solid foundation of social connectedness pre-pandemic may have allowed some of our participants to simply transition from face-to-face to a virtual way of connecting with others, lessening the effects of social isolation.

Other participants recounted having nurtured some of their relationships during the lockdown. One participant explained, “I even reconnected with my aunt because she lives alone, and I didn’t want something to happen to her without me knowing. It’s through social media that I communicate a lot more than before.” Another participant described a similar experience: “One of my friends is a bit older and she’s scared, so we have an email check-in every morning. So, if every morning, we don’t hear back by 10 AM, we call to see what’s going on. Just to be sure we’re okay.” For some people, reaching out to help others motivated them to increase their socialization with family, friends, and acquaintances.

From the interviews conducted by the researchers, it was clear that the experience of pain is not a monolith. The participants ranged across a broad spectrum relative to how much their pain journey and experience had disrupted their pre-pain lives. Lima et al. (2014) explain that the current understanding of chronic pain is an individual experience of psychological, physical, and social factors that are intertwined with the individual who expresses pain. Those who had found and relied on effective para-medical or community management techniques described their pre-COVID life as little disrupted by chronic pain. With the closures created by the pandemic, these participants were no longer able to access their treatments (physiotherapy, massage therapy, etc.) or communities (friends and family, volunteering, gyms, pools, etc.). This caused a major disruption in their daily routines and increased their pain as a result. This finding is consistent with other articles that highlighted that people in quarantine can experience heightened stress and anxiety and an increase in symptoms of depression or post-traumatic stress disorder (Garcovich et al., 2020; Holmes et al., 2020; Sun et al., 2021). On the contrary, other participants did not rely on these management techniques, partly because they had found them ineffective in relieving their pain. These participants considered themselves to be socially isolated even before the pandemic and did not see major changes to their daily life once the social distancing measures were enacted.

### Shrinking Worlds

#### Loss of access to medical care

The origins of the word “care” include sorrow, anxiety, grief, burdens of mind, serious mental attention, as well as to lament and grieve. It also means in a colloquial sense to feel concerned or have an inclination and have a fondness for another (Hovey, 2006). Participants also described various types of loss associated with the consequences of confinement, such as having lost regular medical care, appointments, procedures, or even surgeries due to COVID-19:

What I’ve found difficult during the pandemic was getting appointments because many radiology centers were closed. I widened my search to try to find somewhere that would be open. In the end, it didn’t work so I had to call back the physician who was able to do a consultation over the phone then send a prescription directly to the hospital.

Health care providers did their best to attend to the needs of their chronic pain patients through telephone consultations, among other platforms. Nevertheless, there was a missing element to the healing process—a lack of personal connection—relative to face-to-face interactions:

We’re having trouble seeing our doctors. It’s a problem because we basically have to go to emergency, or we can’t really go.

First, during the pandemic, there were some services we didn’t get. I had a consultation with a doctor, and I couldn’t [get it]. I even had appointments with specialists for [. . .] injection. It didn’t get done.

Missing appointments adds another level of anxiety because of the personal fragility of living with chronic pain under pre-pandemic conditions. People living with chronic pain depend on these treatment modalities for the modification of pain to a level where they can participate in their lives as best as possible:

I was supposed to be operated in early April for a bariatric surgery. Everything was delayed and rescheduled. The expectation I had was to lose weight to help with my situation and my back. [. . .] I was notified ten days before my operation. [. . .] I was really depressed when it happened.

For the person living with pain the “next” new treatment, surgery, nerve block, or medication lends hope that their incarceration due to pain would be lifted and the possibility of relief becomes a powerful motivational force for hope:

It’s been about six months that I’ve been on a waiting list for the pain clinic in Lévis [a suburb of Quebec City, added by authors] to try to help me manage my pain better, but I’ve been told there’s a two-year wait. So now with COVID, is it going to be three years before they start seeing people again? That’s what I’ve found unfortunate.

The disappointment expressed through the participant’s quote is representative of the long wait times, which are explained as extensive and perhaps demoralizing.

These quotes broach varying degrees of loss of access to medical care. Living with chronic pain seems to be living with a whole separate agenda filled with appointments, therapy, check-ups, follow-ups, and other related responsibilities. The resources related to pain treatment and management are limited and time-consuming. This pandemic has left people in the unknown with no clear guidance of when their separate agenda can resume.

For those participants who had built a good routine of self-management before the pandemic hit, restricted access to these self-management tools represented a loss of freedom. One participant discussed it as follows:

I no longer have access to professionals for osteopathy, massage therapy. I no longer have access to regular exercising classes. I no longer have access to the pool when I need it or want it. It’s like grieving the freedom to do what I wanted to do when I wanted to do it, specifically in terms of pain management.

Participants who needed to access medical treatment during the closures described their distress from the challenges of accessing care during that time. Long-awaited surgeries and procedures were postponed indefinitely, long waiting lists were put on pause, and care was rarely accessible outside of emergency departments. The precarity of these participants’ access to medical care, coupled with the uncertainty of the general situation of living through a pandemic, caused the participants stress thereby increasing their low-back pain. In fact, Eccleston et al. (2020) explain that when people living with chronic pain are denied assessment or/and treatment, their condition may worsen significantly.

#### Loss of livinghood

As an example of loss of livinghood, a participant shared that she had a serious car accident in the past and felt anxious around vehicles and busy streets. Being confined to her home exacerbated her reluctance to cross streets that she had been able to cross in the past:

I realized that since I hadn’t been to the gym, I started being afraid to cross . . . I have a small boulevard to cross to go to the gym so even that was difficult every time I would go. I’d tell myself “I crossed it today, good job!” Back and forth and back and forth. But now it’s been almost two months that I haven’t had to go so when I’d go for walks, I’d go the other way. I wouldn’t go toward the boulevard because I didn’t want to face it.

Daily challenges that are taken for granted differ from person-to-person. Something that was challenging pre-pandemic, such as crossing a boulevard, can seem insurmountable during a pandemic. The act of accomplishment and motivation, however insignificant to others, can be challenging for someone living with chronic low back pain and can affect one’s sense of livinghood. Gaining those accomplishments and skills back may be a long journey for those suffering from chronic pain.

Loss of livinghood was also discussed in COVID-19-related patient experience literature (Hovey, 2020; Pavate, 2020; Vigouroux, 2020). Living with chronic pain during this pandemic may seriously disrupt one’s routine and therefore living in an anxiety-inducing situation may add another layer to an already pained existence. Dreyfus explains, “Anxiety reveals the groundlessness of the world and of a person being-in-the-world. The world collapses away from the anxious individual; he or she withdraws because the worldliness of the world is conceptualized” (Dreyfus, 1991). One’s sense of self, which previously defined one’s livinghood, can be vastly altered and disrupted when living during a world-changing event, creating uncertainty, and encompassing psychological distress. In addition, a study investigating the experiences of single women living alone during COVID-19 reported multiple negative experiences that accompany social distancing measures arising from the interrupted flow of daily lives (Kamin et al., 2021). Destabilizing a person living with chronic pain can be debilitating because most rely on a routine to manage their pain.

### Stress

Another important finding from this study was that all participants described an increase of one form or another of stress in their lives since the beginning of the pandemic. Stress can be defined as such: “Stress is a mental and physical response to change in our lives. It is neither negative nor positive, but the response of an individual to a certain situation or experience” (Hovey, 2006).

Some participants described stress related to work, job security, their personal lives, concern for their loved ones, or their own health. This continuum of stress-related experiences led many participants to discuss an increase in pain. Here are several examples of participants expressing the stress related to their chronic low back pain within the context of the pandemic:

To imagine myself sick with the condition I have . . . I’d really just be home and I can’t see how I would manage all this on top of the pain.

The amount of work increased; stress also increased. So, since the beginning of the pandemic, I’ve had an increase in my low-back pain.

There are a lot of disinfection procedures and so it keeps me from being tuned into the warning signs of greater pain. I’m so focused on what not to forget that sometimes I don’t realize that I’m in pain, but given the intensity of the pain, I should have reacted before . . . but my mind wasn’t available.

I noticed that low back pain with stress is not easy. Stress increases pain. If you don’t feel good in your head, you won’t feel good. You have more perceptions.

I work in healthcare. I’m a respiratory therapist. The first thing that happened at work was that panic started. Stress levels increased.

Job insecurity means that, since my medication is extremely expensive and my insurance pays for it, it causes me extra stress.

There was definitely a financial stress. I came back [to Canada, added by authors] before the confinement and I had to start working but my hiring was temporarily suspended.

This widespread increase in stress, which led to an intensification in pain for people living with chronic pain, happened at a time when medical professionals were difficult to reach. The compounded effect of experiencing stress and pain, and not having access to usual coping mechanisms or medical professionals, was distressing to participants.

Another point to highlight is that, while the participants did not consider themselves more at risk of contracting the COVID-19 virus, they feared that their experience of being sick with the virus would be made even more difficult due to their chronic pain. This anticipated vulnerability of being sick while living with chronic pain created additional stress for many of the participants. Gadamer writes, “Anxiety is intimately connected with an oppressive sense of constriction, with sudden exposure to the vastness and strangeness of the world” (Gadamer, 1996). The experience for the person becoming the patient with the illness itself is the need to unlearn one’s way of life and then relearn how to navigate a new way of being in the world (Hovey, 2018).

However, not all the effects of the COVID-19 pandemic on this population were negative. Some participants, particularly those who had grown accustomed to living an isolated life, were delighted that many of their loved ones were confined safely in their homes. This fostered an era of virtual socialization, which was more accessible to our participants than in-person socialization as it did not require commuting or uncomfortable seating arrangements. The participants who may not have socialized much before the pandemic due to busy schedules saw their socialization levels and connectedness to family and friends increase because of the many virtual platforms available to them.

### Expectations of the Future

During the interviews, participants were often hesitant to visualize themselves in the future. They explained that during their chronic pain journey, they had to learn to live on a day-to-day basis. Making plans for the following day could sometimes be challenging given the unpredictable fluctuations in their pain. The pandemic and its associated confinement and social distancing measures emphasized the participants’ hesitations to envision the future. Instead, they preferred to focus on the day at hand and manage their expectations daily:

I try not to have to many expectations for the future. Living with chronic pain unfortunately took “looking at the future” away from me. I try to look more towards every day.

It’s really hard to project yourself into the future. It’s hard because I see no way out right now.

It’s hard to see further than tomorrow, to even survive the day because of the pain.

It’s easier not to project into the future so we’re not disappointed.

One participant did try to project herself into the future:

Some dreams are crumbling because of the pandemic. When you think about your next trip at 66 years old, you tell yourself that you won’t be able to travel until they find a vaccine, so it’ll take a while. Without being depressed, it takes away a little bit of . . . spice from life sometimes.

**Figure 2.**
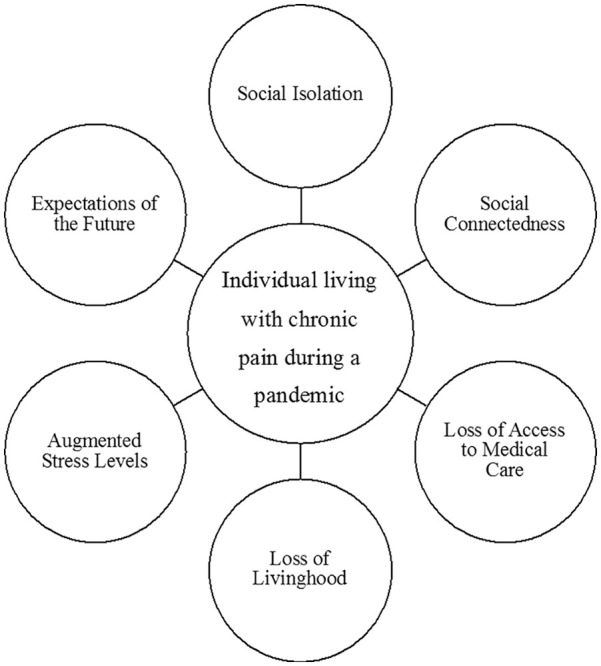
Summary of findings of an individual living with chronic pain during a pandemic.

Our findings (Figure 2) indicate that participants were reluctant to project themselves into the future, explaining that living in the now is emotionally safer. When current concerns are extended into the future, they can hold feelings of fear and hope, within a context that may or may not occur. This projection affects the now, through expectations (Jones & Chapman, 2000). Projections based on desirable results create hopeful expectations, such as future desirable plans, which are valued and positive. Projections of undesirable outcomes, on the contrary, evoke fearful expectations, like awaiting news that can be potentially life changing and disruptive to our being-in-the-world (Gullickson, 1993; Jones & Chapman, 2000; Margaretha Strandmark, 2004; Sheehan, 1998). Both sides of this coin can lead to suffering as they either depict an unfulfilled positive expectation or an undesirable expectation.

## Discussion

Some of the experiences described in our findings are not unique to people living with chronic pain and can be transferred to the general population. Under a similar context, a study examining the psychological and coping responses from previous infectious disease outbreaks, such as the Ebola epidemic and the H1N1 outbreak, has demonstrated common findings within the general population. Examples of these include anxiety, fear, depression, anger, guilt, grief, loss, and post-traumatic stress as well as a deepening compassion toward others (Chew et al., 2020). The findings detailed by Chew et al. echo the findings in the present study, despite the difference in the study population. However, this has not been specifically addressed in a low back chronic pain population.

### Recommendations

All the findings and quotes in this study come directly from participants living with chronic pain and must therefore be examined as such. While some findings are transferrable to the general population, others are more specific to people with chronic pain, such as the need for continued access to regular health care. This may include access to their family physician and specialists to ensure continued access to prescriptions, care, support, and advice during the ongoing pandemic. This care is even more important given the challenging nature of the measures taken to slow the spread of COVID-19. One way to ensure continued access to health care is through telehealth. Telehealth is defined as the delivery and facilitation of health-related services via telecommunications technologies. Introducing remote supported pain management services can increase the accessibility and scalability of services offered for people living with chronic pain (Eccleston et al., 2020). For context, it is important to understand that telehealth was not widely used in Quebec prior to the COVID-19 pandemic and was not widely or readily available during the “first” wave, when the interviews were conducted. Understandably, not all care interventions can be achieved through telehealth. For example, due to their nature, para-medical interventions such as physiotherapy and massage therapy require in-person appointments and physical proximity between client and practitioner. Our findings suggest that allowing these practitioners to remain operational, even during a pandemic, could be beneficial to people living with chronic pain. Health authorities could emit specific safety and sanitary guidelines, to which para-medical practitioners must adhere to remain in operation (Hovey et al., 2020).

### Future Research

Other studies have shown the effectiveness of telehealth as it has emerged over the past year; however, more research is needed to understand its implications and limitations for people living with chronic pain (Eccleston et al., 2020). This article also invites longitudinal studies on the social aspects for people living with chronic pain during the pandemic and to proactively help them gain access to meaningful groups to enhance their social connectedness.

### Conclusion

In many of our conversations with participants, uncertainty was most often discussed regarding their health, the length of the confinement measures, the nature and spread of the virus, and concern for self and others. The strict confinement measures put into place by the Quebec provincial government also caused the closing of many health care services, which affected the participants’ ability to access the services they relied on for the management of their chronic low-back pain. In future “waves,” it will be important for public health officials to remember the needs of people living with chronic pain in their closure decisions. Ensuring access to health care, even during future “waves” of the pandemic, will help mitigate the stress caused by the uncertainty felt by so many participants in this project. The multifaceted nature of chronic pain as a health condition demands that people who live with this condition have access to their health care providers beyond just emergency rooms and pharmacies.
